# Identification and Validation of ATF3 Serving as a Potential Biomarker and Correlating With Pharmacotherapy Response and Immune Infiltration Characteristics in Rheumatoid Arthritis

**DOI:** 10.3389/fmolb.2021.761841

**Published:** 2021-12-13

**Authors:** Huan Hu, Facai Zhang, Li Li, Jun Liu, Qin Ao, Ping Li, Jiashun Zeng, Long Li

**Affiliations:** ^1^ Department of Rheumatology and Immunology, The Affiliated Hospital of Guizhou Medical University, Guiyang, China; ^2^ Department of Urology, The Affiliated Hospital of Guizhou Medical University, Guiyang, China; ^3^ Medical Intensive Care Unit, The Affiliated Hospital of Guizhou Medical University, Guiyang, China; ^4^ Department of Cardiology, The Affiliated Hospital of Guizhou Medical University, Guiyang, China

**Keywords:** ATF3, biomarker, rheumatoid arthritis, immune infiltration, Gene Expression Omnibus

## Abstract

**Background:** Although disease-modifying antirheumatic drugs (DMARDs) have significantly improved the prognosis of patients with rheumatoid arthritis (RA), approximately 40% of RA patients have limited response. Therefore, it was essential to explore new biomarkers to improve the therapeutic effects on RA. This study aimed to develop a new biomarker and validate it by an *in vitro* study.

**Methods:** The RNA-seq and the clinicopathologic data of RA patients were downloaded from Gene Expression Omnibus (GEO) databases. Differentially expressed genes were screened in the GPL96 and GPL570 databases. Then, weighted gene co-expression network analysis (WGCNA) was used to explore the most correlated gene modules to normal and RA synovium in the GPL96 and GPL570 databases. After that, the differentially expressed genes were intersected with the correlated gene modules to find the potential biomarkers. The CIBERSORT tool was applied to investigate the relationship between activated transcription factor 3 (ATF3) expression and the immune cell infiltration, and Gene Set Enrichment Analysis (GSEA) was used to investigate the related signaling pathways of differentially expressed genes in the high and low ATF3 groups. Furthermore, the relationships between ATF3 expression and clinical parameters were also explored in the GEO database. Finally, the role of ATF3 was verified by *in vitro* cell experiments.

**Results:** We intersected the differentially expressed genes and the most correlated gene modules in the GPL570 and GPL96 databases and identified that ATF3 is a significant potential biomarker and correlates with some clinical–pharmacological variables. Immune infiltration analysis showed that activated mast cells had a significant infiltration in the high ATF3 group in the two databases. GSEA showed that metabolism-associated pathways belonged to the high ATF3 groups and that inflammation and immunoregulation pathways were enriched in the low ATF3 group. Finally, we validated that ATF3 could promote the proliferation, migration, and invasion of RA fibroblast-like synoviocyte (FLS) and MH7A. Flow cytometry showed that ATF3 expression could decrease the proportion of apoptotic cells and increase the proportion of S and G2/M phase cells.

**Conclusion:** We successfully identified and validated that ATF3 could serve as a novel biomarker in RA, which correlated with pharmacotherapy response and immune cell infiltration.

## Introduction

Rheumatoid arthritis (RA) is a chronic autoimmune disease characterized by synovitis and joint destruction, which affects approximately 1% of the population in the world (Smolen et al., 2016; Myasoedova et al., 2020). The pathogenesis of RA is involved in lesions on the synovial membrane, which are accompanied by synovial hyperplasia and inflammation. The synovium contains mesenchymal-derived fibroblast-like synoviocytes (FLSs) and resident macrophages. FLSs, a key component of invasive pannus, play an important role in the pathogenesis of RA (Alsaleh et al., 2011; Quirke et al., 2011; Nygaard and Firestein, 2020). RA-FLS could proliferate and secrete a variety of immunomodulatory factors, vascular endothelial growth factor (VEGF), and matrix metalloproteinases (MMPs) (Liang et al., 2020). Although disease-modifying antirheumatic drugs (DMARDs) have significantly improved the prognosis of patients with RA, approximately 40% of RA patients have a poor response and suffer from pain and loss of daily living activities, which not only increase the financial burden of RA patients but also expose patients to potential therapeutic side effects (Humby et al., 2021). Therefore, it is essential to explore new biomarkers to provide a theoretical possibility for the development of new targeted drugs.

It is due to the significant role of FLSs in the pathogenesis of RA that we explored the potential biomarkers in the synovium, which is rich in FLSs and easy to analyze using next-generation sequencing (NGS). We first screened out the differentially expressed genes by comparing the RNA-seq between normal and RA synovium. Then, we used weighted gene co-expression network analysis (WGCNA) to explore the most correlated gene module with normal and RA synovium. After that, we intersected the differentially expressed genes with the gene module to explore potential biomarkers, and we finally identified that activated transcription factor 3 (ATF3) might be a significant biomarker worthy of verification by *in vitro* cell function experiments.

ATF3, consisting of 181 amino acids with a molecular weight of 22 kDa, belongs to the protein family of ATF/cAMP response element binding (CREB) transcription factors, shares a common basic-region leucine zipper (bZIP) element, and is bound to the target DNA through the basic region in this domain (Liang et al., 1996; Hai et al., 1999; Thompson et al., 2009). An appropriate ATF3 activity is important for the normal physiology of cells, and ATF3 dysfunction is also related to various pathophysiological responses, such as inflammation, apoptosis, oxidative stress, and endoplasmic reticulum stress (Liang et al., 1996; Hai et al., 1999; Thompson et al., 2009). Although accumulating evidence showed that ATF3 plays a critical role in fibroblast cell activation in various diseases (Kim et al., 2017; Wu et al., 2019; Zu et al., 2020), there is still no study that explores ATF3 in RA-FLS. Therefore, we aimed to explore the role of ATF3 in RA by *in vitro* cell experiments and investigate its correlation with pharmacotherapy response and immune infiltration characteristics via the bioinformatics method.

## Materials and Methods

### Data Collection

Four RA-associated microarrays (GSE12021, GSE55235, GSE55457, and GSE55584) in the GPL96 platform were downloaded from Gene Expression Omnibus (GEO). Then RNA-seq data from RA and normal synovial tissues in the four microarrays were extracted and merged, and batch effect was reduced by the ComBat package in R software (www.datavis.ca/R/). Similarly, GSE48780 and GSE77298 were also retrieved from the GPL570 platform, and the transcriptome data were downloaded and merged before the batch effect was reduced. Moreover, RNA-seq and clinical data in GSE13026, GSE21537, and GSE45867 were also used to explore the relationships between ATF3 and RA status and the pharmacotherapy response.

### Identification of Differentially Expressed Genes, Function Enrichment Analysis, and Protein–Protein Interaction Network Construction

The “edgeR” package in R software was used to screen out the differentially expressed genes with **|**log Fold-Change| ≥ 1 and false discovery rate (FDR) **<**0.05 in the GPL96 and GPL570 databases. After that, Gene Ontology (GO) enrichment analysis and Kyoto Encyclopedia of Genes and Genomes (KEGG) pathway analysis were performed to investigate enriched potential function and pathways in these differentially expressed genes with the “clusterProfiler” package in R software. Moreover, the differentially expressed genes were also uploaded to the STRING website (www.string-db.org) to visualize the function protein association network with a minimum required interaction score larger than 0.99.

### Weighted Gene Co-Expression Network Analysis Construction and Identification of Key Modules Associated With Rheumatoid Arthritis Samples

WGCNA, a scale-free network algorithm based on gene expression microarray data, is widely used for finding clusters of highly correlated genes in bioinformatics applications. First, the genes were screened with SD larger than 0.5 among all samples and were imported to the co-expression network. In view of data integrity and little batch effect in the GPL96 and GPL570 databases, the sample trees were constructed with cut height = 2,000, and the selected samples were applied to build the sample dendrogram and clinical trait heatmap. Second, the pickSoftThreshold function in the “WGCNA” package in R software was used to estimate an optimum soft threshold power, which struck a balance between higher *R* ^2^ and higher mean connectivity. Third, the adjacency values among the imported genes were calculated according to the optimum soft threshold power in both databases and were transformed to topological overlap matrix (TOM). Furthermore, the corresponding dissimilarity (1 − TOM) was also calculated, which was significant for hierarchical clustering of genes. Fourth, modules were identified with the dynamic tree cut method by hierarchical clustering of genes, which used dissimilarity (1 − TOM) as distance measurement with module size larger than 50 and deep split = 2. Fifth, the most clinically relevant module was selected, and the module membership (MM) and gene significance (GS) were calculated. After that, the two differentially expressed gene sets and two clinically relevant modules in the GPL96 and GPL570 databases were intersected, and it was found that ATF3 was a differentially expressed clinical-relevant gene, which might be a potential biomarker worthy of validation by *in vitro* cell experiments.

### Correlation of ATF3 Expression With Clinical Parameters

The transcriptome data and clinical data in GSE13026, GSE21537, and GSE45867 were downloaded to explore the relationships between ATF3 expression and RA status, lymphocyte aggregation, inflammation status, infliximab response, tocilizumab response, methotrexate response, joint location, age, and gender. In the light of the median expression of ATF3, all patients in the microarrays were divided into two groups, and then the clinical parameters of both the high and low ATF3 expression groups were compared. Moreover, ATF3 expression was also compared among groups classified by clinical parameters.

### Immune Infiltration Analysis and Gene Set Enrichment Analysis

All transcriptome data in the GPL96 and GPL570 databases were normalized before being imported to the CIBERSORT tool to estimate the contents of 22 human immune cells in each RA patient. Then, the difference of infiltrating immune cells in the high and low ATF3 expression groups was observed and compared. A *p* < 0.05 was considered statistically significant.

Moreover, the RNA-seq data in both the high and low ATF3 expression groups were uploaded to Gene Set Enrichment Analysis (GSEA) to explore the differentially expressed gene-related signaling pathways. The enriched pathways were screened out based on an FDR < 0.25 and *p* < 0.05 after 1,000 permutations, and the top 10 enriched pathways in both groups were presented in multi-GSEA plots.

### Cell Culture and Infection

Human RA cell lines RA-FLS and MH7A (Beijing Beina Chuanglian Biotechnology Institute, Beijing, China) were cultured in Dulbecco’s Modified Eagle Medium/nutrient mixture F-12 (DMEM/F12; Gibco, Grand Island, NY, USA) supplemented with 10% heat-inactivated fetal bovine serum (FBS; Gibco, USA; 1% penicillin/streptomycin) in a humidified incubator containing 5% CO_2_ at 37°C according to American Type Culture Collection suggestions.

Cells were added to the 6-well plate at a density of 1 × 10^6^/well and incubated for 24 h. Then, a fresh medium containing lentivirus and 8 μg/ml of polybrene [OBiO Technology (Shanghai) Corp., Ltd., Shanghai, China] was added. The addition amount of lentivirus was calculated according to the required multiplicity of infection (MOI). Preliminary experiments determined that the optimal MOI for lentivirus of short hairpin (Sh)-ATF3 (Sh-ATF3), the lentivirus of control (Sh-Ctrl), ATF3-overexpression (ATF3-OE), and control lentivirus (Ctrl-OE) was 100. The culture medium was changed within 24 h after infection, and 1‰ of puromycin (Sigma, St. Louis, MO, USA) was added to the complete medium to kill the wild-type RA-FLS and MH7A. About 14 days later, the majority of the wild-type cells died. The efficiency of infection was determined by quantitative real-time PCR.

### Quantitative Real-Time PCR

The gene expression levels of Sh-ATF3 and ATF3-OE were evaluated by qRT-PCR. Total RNA was isolated using Invitrogen TRIzol reagent (Thermo Fisher Scientific, Waltham, MA, USA) according to the manufacturer’s instructions. The isolated total RNA was then reverse transcribed into cDNA using the QuantScript RT cDNA Synthesis Kit (Bio-Rad, Hercules, CA, USA). The Maxima SYBR Green qPCR kit (Thermo Fisher Scientific, USA) was used for qRT-PCR analysis. The oligonucleotide primer sequences including forward and reverse used in these experiments were presented as follows: ATF3: 5′-CCT​CTG​CGC​TGG​AAT​CAG​TC-3′ and 5′-TTC​TTT​CTC​GTC​GTC​GCC​TCT​TTT​T-3′, IL-1β: 5′-GTC​GGA​GAT​TCG​TAG​CTG​GA-3′ and 5′-GTC​GGA​GAT​TCG​TAG​CTG​GA-3′, IL-6: 5′-ACT​CAC​CTC​TTC​AGA​ACG​AAT​TG-3′ and 5′-CCA​TCT​TTG​GAA​GGT​TCA​GGT​TG-3′, IL-8: 5′-ACT​GAG​AGT​GAT​TGA​GAG​TGG​AC-3′ and 5′-AAC​CCT​CTG​CAC​CCA​GTT​TTC-3′, and β-actin: 5′-CAT​GTA​CGT​TGC​TAT​CCA​GGC-3′ and 5′-CTC​CTT​AAT​GTC​ACG​CAC​GAT-3′. The relative mRNA level was calculated by the 2^−ΔΔCt^ method.

### Cell Proliferation Assay

Stable cell lines were constructed using the ATF3 lentivirus. The cells were plated into 96-well plates at 1 × 10^3^ cells/well. Cell viability was measured at 1, 2, 3, and 4 days using Cell Counting Kit-8 (CCK-8) reagent (96992; Sigma, St. Louis, MO, USA) according to the manufacturer’s instructions. Optical density (OD) was measured at 450 nm using an ELx800 Absorbance Microplate Reader (BioTek, Winooski, VT, USA).

### Cell Cycle Assay

RA-FLS and MH7A were detached as single-cell suspension after 48-h transfection and fixed in 75% ethanol overnight at 4°C. Fixed cells were washed and stained with 25 mg/ml of propidium iodide (PI; Sigma, USA) in phosphate-buffered saline (PBS) containing 0.1% Triton and 10 mg/ml of RNase (Thermo Fisher Scientific, USA) on ice for 0.5 h in the dark. The cell cycle was analyzed by flow cytometry on a FACSCalibur system (BD Biosciences, San Jose, CA, USA). The percentage of cells in different cell cycle phases was calculated by ModFit 3.0 (Verity Software House, Inc., Topsham, ME, USA).

### Apoptotic Assay

The Annexin V-APC/7-AAD kit (Multisciences, Hangzhou, China) was used to visualize apoptotic cells according to the manufacturer’s instructions. RA-FLS and MH7A were infected with Sh-ATF3, Sh-Ctrl, ATF3-OE, and Ctrl-OE lentiviruses. Cells were washed with PBS and recombined in 1× binding buffer, then treated with Annexin VAPC and 7-AAD for 15 min at room temperature (RT) in the dark, and then analyzed by flow cytometry.

### Migration and Invasion Assay

Cell migration and invasion (Matrigel added) were evaluated using the transwell assay (Corning, New York, NY, USA). Cells (4 × 10^4^ cells/well for migration and invasion assay of ATF3 knockdown and 2 × 10^4^ cells/well for migration and invasion assay of ATF3 overexpression) in serum-free DMEM were seeded into the upper chamber (pre-coated with Matrigel (R&D Systems, Minneapolis, MN, USA) for invasion assay), and media containing 10% FBS were added into the lower chamber. After being incubated for 24 h, the cells were fixed with 70% ethanol, stained with 0.1% crystal violet, and photographed under an inverted microscope. The number of cells in five random fields per well was calculated, and the average value was taken as the migration and invasion numbers.

### Measurement of Inflammatory Cytokine Release

Stable infection of RA-FLS and MH7A was treated with or without 10 ng/ml of tumor necrosis factor-α (TNF-α) (Propotec, R&D Systems, Minnesota, USA) for 24 h, and the supernatants were collected to determine the levels of IL-1β, IL-6, and IL-8 with ELISA kits.

### Statistical Analysis

All statistical analyses were performed in the R software and GraphPad Prism 9. Quantitative data of two groups were compared by Student’s *t*-test, and quantitative data of three or more groups were compared by one-way ANOVA or Welch’s test. A *p* < 0.05 was regarded as statistically significant.

## Results

### Selection of Differentially Expressed Genes

**FIGURE 1:**
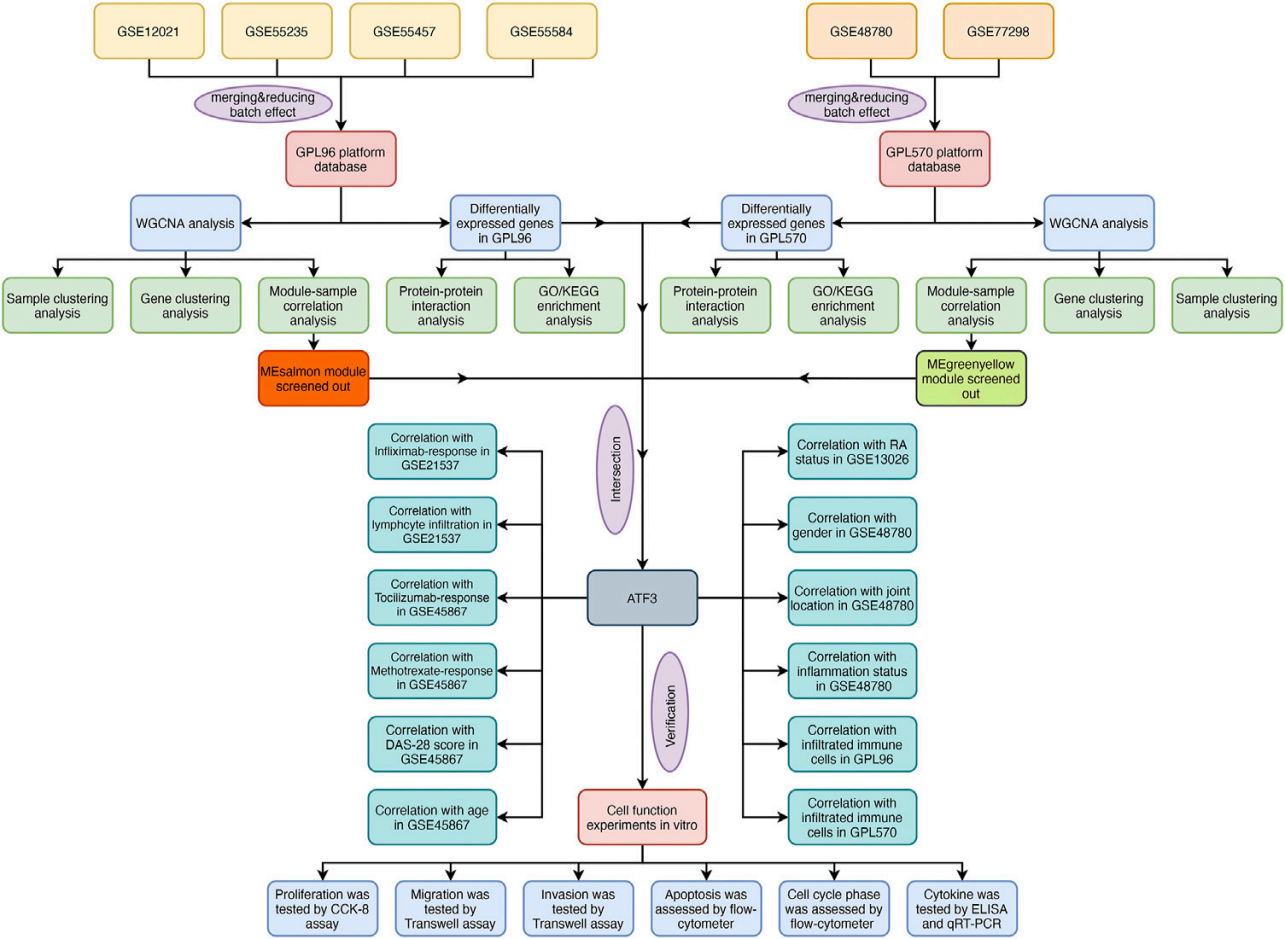
The flowchart of the analysis.

**FIGURE 2:**
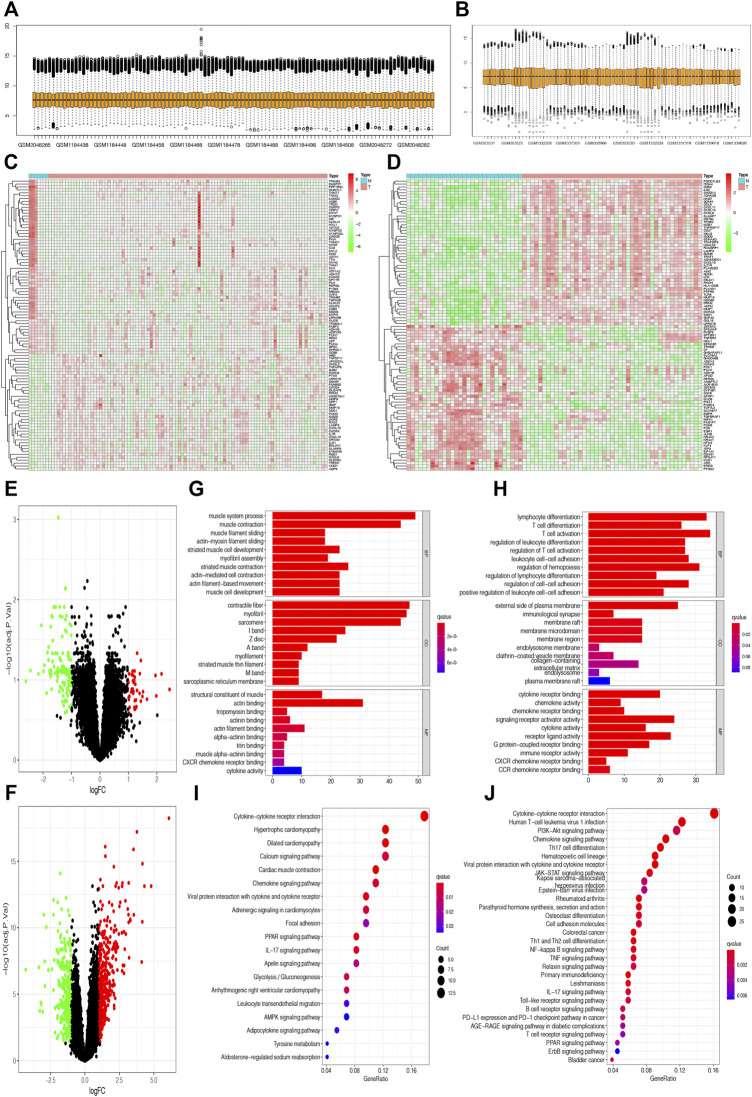
Selection of differentially expressed genes and enrichment of related pathways. **(A and B)** Gene expression in different samples was normalized in the GPL570 and GPL96 databases. **(C and D)** The heatmaps show the top 50 differentially expressed genes between synovial tissues and rheumatoid arthritis (RA) tissues in the GPL570 and GPL96 databases. **(E and F)** The volcano plots show the differentially expressed genes with false discovery rate (FDR) <0.05 and |log Fold-Change| ≥ 1 in the GPL570 and GPL96 databases. **(G and H)** The Gene Ontology (GO) enrichment analysis based on differentially expressed genes was performed in the GPL570 and GPL96 databases. **(I and J)** The Kyoto Encyclopedia of Genes and Genomes (KEGG) enrichment analysis was performed to explore the associated pathways in the GPL570 and GPL96 databases.

The flowchart shows all essential and significant procedures in our study (Figure 1). The transcriptome data in GSE12021, GSE55235, GSE55457, and GSE55584 were downloaded and merged as the GPL96 database. Similarly, the RNA-seq data in GSE48780 and GSE77298 were also acquired and merged as the GPL570 database. The ComBat function was used to reduce batch effect among different microarrays in the two merged databases, and the normalizeBetweenArrays function was used to normalize gene expression among different samples in the GPL570 and GPL96 databases (Figures 2A,B). After data normalization, the heatmaps showed the top 50 differentially expressed genes between synovial tissues and RA tissues (Figures 2C,D). Meanwhile, the volcano plots showed that there were 163 and 707 differentially expressed genes with FDR < 0.05 and **|**log Fold-Change**|** ≥ 1 in the GPL570 and GPL96 databases, respectively (Figures 2E,F).

### Function Enrichment Analysis and Construction of Protein–Protein Interaction Network

We used the “clusterProfiler” package in R software to explore the potential GO and KEGG function enrichment in those differentially expressed genes. The GO enrichment analysis showed that those differential genes in the GPL570 database were significantly enriched in the muscular system process, muscle contraction in the subset of biological process (BP); enriched in contractile fibers, myofibrils in the subset of cell compartment (CC); and enriched in the structural constituent of muscle, actin binding in the subset of molecular function (MF) (Figure 2G). Meanwhile, the GO enrichment analysis showed that those differential genes in the GPL96 database were significantly enriched in immune- and inflammation-associated functions, such as lymphocyte differentiation, T-cell differentiation in the subset of BP; external side of the plasma membrane, immunological synapse in the subset of CC; and cytokine receptor binding, chemokine activity in the subset of MF (Figure 2H).

Moreover, KEGG enrichment analysis showed that those differentially expressed genes in the two databases were all significantly enriched in some inflammation-associated pathways, such as cytokine–cytokine receptor interaction, chemokine signaling pathway, viral protein interaction with cytokine and cytokine receptor, and IL-17 signaling pathway (Figures 2I,J), which were in accordance with inflammatory attributes of RA.

**FIGURE 3:**
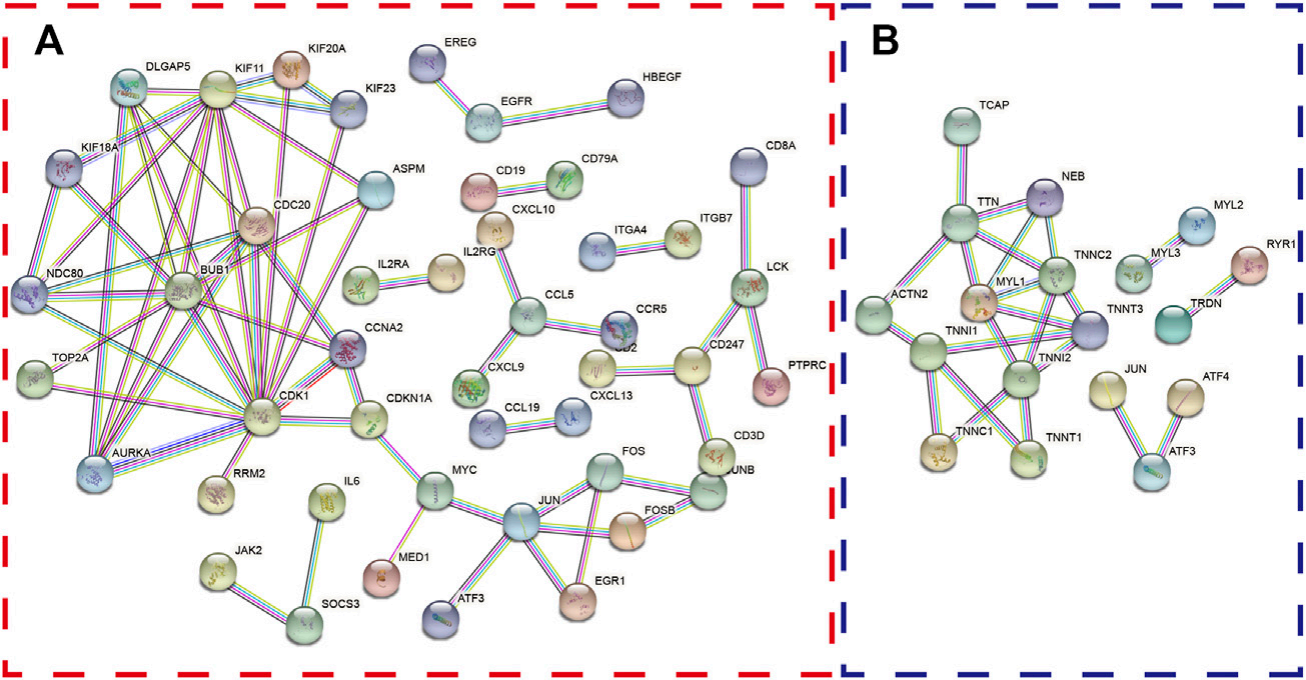
The protein–protein interaction (PPI) network was constructed with differentially expressed genes. The PPI network shows the interactions among genes with a minimum required interaction score >0.99 in the GPL96 database **(A)** and GPL570 database **(B)**.

Furthermore, the differentially expressed genes in the two databases were also uploaded to STRING website to explore the protein–protein interaction (PPI). Interestingly, we found that ATF3 was incorporated into the two PPI networks with a minimum required interaction score larger than 0.99 and had close relationships with JUN and ATF4 (Figures 3A,B).

### Weighted Gene Co-Expression Network Analysis Construction and Identification of Key Modules Associated With Rheumatoid Arthritis

**FIGURE 4:**
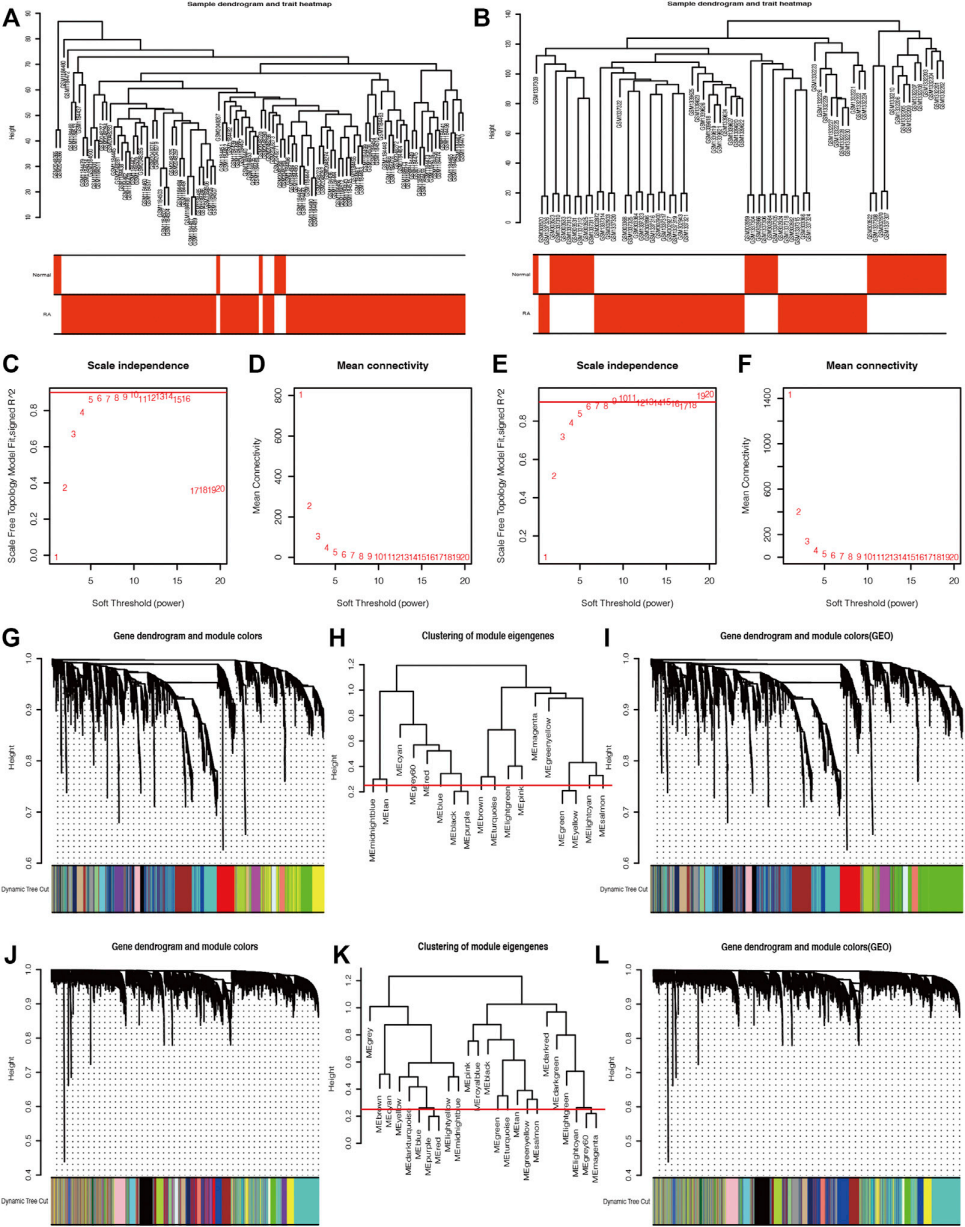
The weighted gene co-expression network analysis (WGCNA) algorithm was applied to explore the most correlated gene modules. The sample dendrogram and trait heatmaps of the GPL96 database **(A)** and GPL570 database **(B)**. The optimum soft threshold power was determined based on the scale independence plot and mean connectivity plot in the GPL570 **(C and D)** and GPL96 databases **(E and F)**. The gene clustering dendrogram was constructed based on dissimilarity, and the related gene modules were merged with the dynamic tree cut method by hierarchical clustering in the GPL570 **(G–I)** and GPL96 databases **(J–L)**.

One hundred six samples, including seven normal synovial tissues and 99 RA synovia tissues, in the GPL570 database were presented in the sample dendrogram and trait heatmap with cut height = 2,000 in order to ensure data integrity (Figure 4A). Similarly, the sample dendrogram and trait heatmap showed that 29 normal synovial tissues and 45 RA synovia tissues were included in the GPL96 database (Figure 4B). The pickSoftThreshold function in the “WGCNA” package was applied to estimate optimum soft threshold powers, which could strike a balance between *R* ^2^ and mean connectivity and corresponded to five and six in the GPL570 and GPL96 databases, respectively (Figures 4C–F). After the soft threshold power was chosen, the adjacency value was calculated among the selected genes with SD > 0.5 and was transformed to TOM. The gene clustering dendrogram was constructed according to TOM-based dissimilarity, and the gene modules were identified and merged with the dynamic tree cut method (cut height = 0.25) by hierarchical clustering of genes in the GPL570 and GPL96 databases (Figures 4G–I and J–L).

**FIGURE 5:**
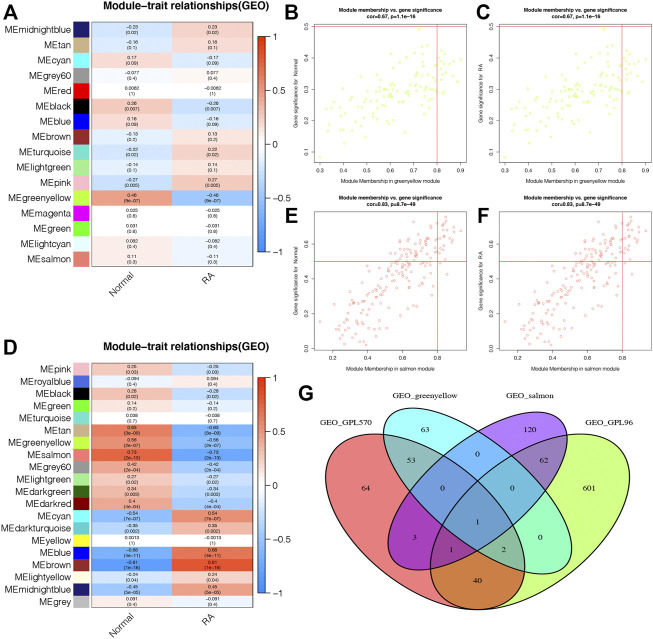
The selection of potential biomarkers based on the most correlated gene modules and differentially expressed genes. **(A and B)** The module–trait relationship plots shows that ME-greenyellow module and ME-salmon module were the most correlated gene modules in the GPL570 and GPL96 databases, respectively. The genes in the ME-greenyellow module and ME-salmon module had a significant correlation with normal and rheumatoid arthritis (RA) samples in the GPL570 **(C and D)** and GPL96 databases **(E and F)**. **(G)** The ME-greenyellow module genes, the ME-salmon module genes, and differentially expressed genes in the GPL570 and GPL96 databases were intersected to screen out final potential biomarkers.

The module–trait correlation heatmap showed that the ME-greenyellow module was the most positively correlated with normal synovial tissues and was the most negatively correlated with RA synovial tissues (Figure 5A). Meanwhile, the module–trait correlation heatmap of GPL96 indicated that ME-salmon module had the most positive correlation with normal samples and the most negative correlation with RA samples (Figure 5B). Moreover, the correlation plots between MM and GS showed that the genes in the ME-greenyellow module and ME-salmon module had a significant correlation with normal and RA samples and could be regarded as a potential set of biomarkers (Figures 5C–F).

Finally, we intersected the ME-greenyellow module genes and differentially expressed genes in the GPL570 database and the ME-salmon module genes and differentially expressed genes in the GPL96 database, and we found that only one gene, ATF3, was screened out (Figure 5G).

### Relationship Between ATF3 Expression and Clinical Parameters

In addition to the transcriptome and associated clinical information of the GPL570 and GPL96 databases, the RNA-seq and clinical data in GSE13026, GSE21537, and GSE45867 were also downloaded to investigate the relationships between ATF3 expression and clinical parameters, such as age, gender, joint location, RA status, lymphocyte aggregation status in the synovium, inflammation status in the synovium, infliximab response, tocilizumab response, and methotrexate response. All patients in these microarrays were divided into the high and low ATF3 groups in the light of the median expression of ATF3.

**FIGURE 6:**
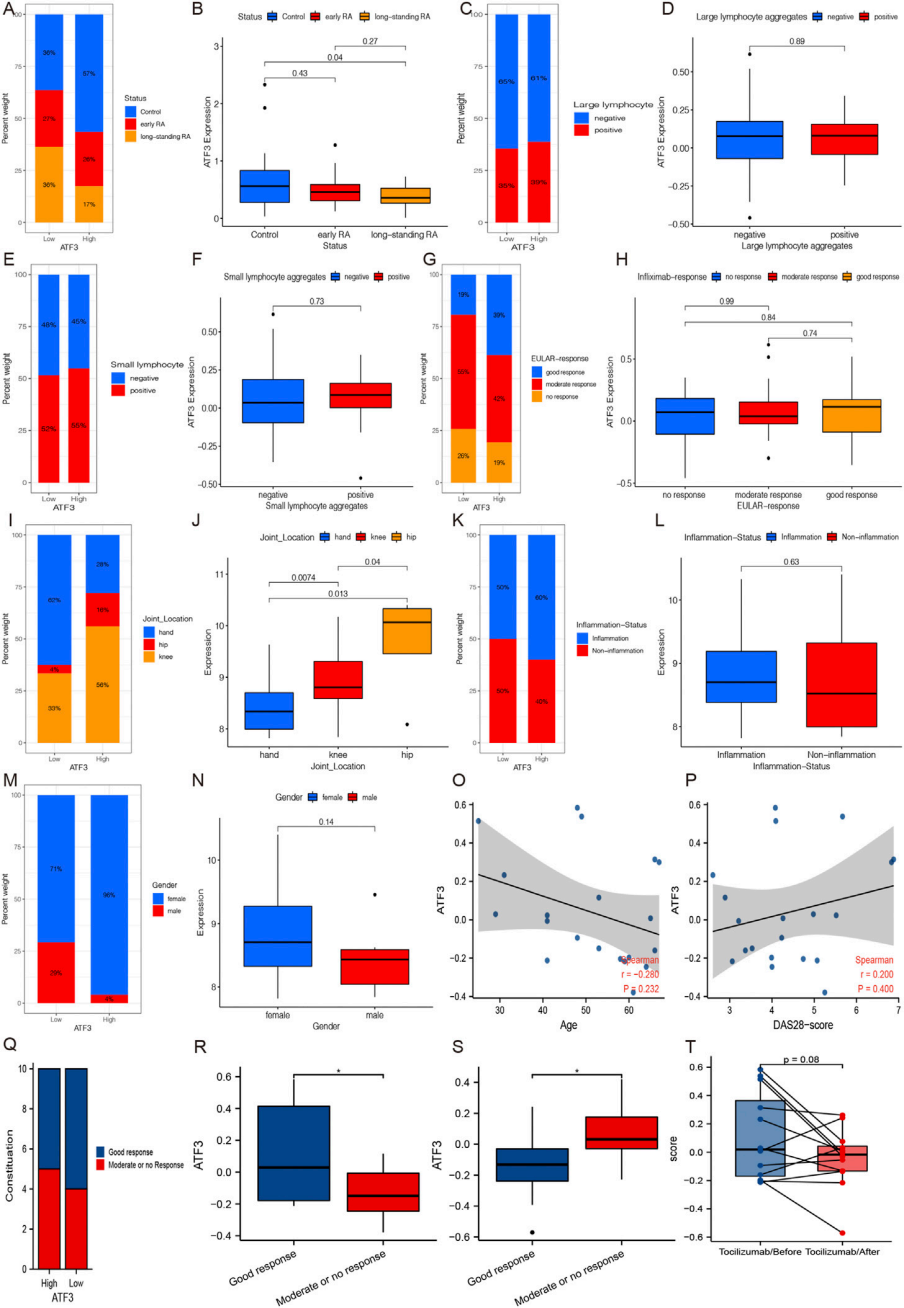
The relationships between ATF3 and clinical parameters. **(A and B)** GSE13026 shows that ATF3 in long-standing rheumatoid arthritis (RA) patients is significantly lower than that in normal individuals, and its expression in long-standing RA patients seemed lower than that in early RA patients. **(C and D)** Large lymphocyte aggregation status in synovium seemed to have no relationships with ATF3 expression in GSE21537. **(E and F)** Similarly, small lymphocyte aggregation status still has no relationship with ATF3 in GSE21537. **(G and H)** Patients with good infliximab response accounted for a higher percentage in the high ATF3 group, but the difference of ATF3 among different infliximab-response groups was not significant. **(I and J)** GSE48780 indicates that ATF3 expression was significantly different among different joint synovium and its expression in hip synovium was significantly higher than that in knees and hands. **(K and L)** ATF3 in inflammation-infiltrated synovium was slightly higher than that in non-inflammation synovium, and patients with inflammation-infiltrated synovium accounted for a higher percentage in the high ATF3 group. **(M and N)** ATF3 expression seemed higher in female patients in GSE48780. ATF3 had a slight decrease along with the increment of age **(O)**, while there was a positive correlation between ATF3 and DAS-28 score **(P)**, an index to evaluate RA activity. **(Q and R)** ATF3 expression in RA patients with a good response was significantly higher than that in patients with a limited response before treatment with tocilizumab or methotrexate. **(S and T)** ATF3 expression in RA synovium after treatment was significantly lower in patients with good response, especially treatment with tocilizumab.

In GSE13026, we found that ATF3 expression in long-standing RA patients is significantly lower than that in normal individuals, and its expression in long-standing RA patients was lower than that in early RA patients. Similarly, there was a higher proportion of normal synovium in the high ATF3 expression group (Figures 6A,B).

Figures 6C–F show that the lymphocyte aggregation status in the synovium seemed to have no relationships with ATF3 expression in GSE21537. Moreover, the clinical data in GSE21537 indicated that cases with good infliximab response accounted for a higher percentage in the high ATF3 group than in the low ATF3 group, although patients with good infliximab response just had a slightly higher ATF3 expression than those with no or moderate response (Figures 6G,H).

GSE48780 in the GPL570 database indicated that ATF3 expression was significantly different among different joint synovium and its expression in hip synovium was significantly higher than that in the knees and hands (Figures 6I,J). Furthermore, ATF3 expression in inflammation-infiltrated synovium was slightly higher than that in non-inflammation synovium, and patients with inflammation-infiltrated synovium accounted for a higher percentage in the high ATF3 group (Figures 6K,L). After that, in mining the clinical data of GSE48780, we also found that ATF3 expression seemed higher in female patients, although the difference between genders was not significant (Figures 6M,N).

In GSE45867, we explored the relationship between ATF3 and age, and we found that ATF3 had a slight decrease along with the increment of age (Figure 6O). Meanwhile, we discovered a positive correlation between ATF3 and DAS-28 score (Figure 6P), which was a significant index to evaluate RA activity and is widely used in clinical practice. Moreover, we also discovered that ATF3 expression in RA patients with a good response was significantly higher than that in patients with a limited response before treatment with tocilizumab or methotrexate (Figures 6Q,R). More interestingly, ATF3 expression in RA synovium after treatment with tocilizumab or methotrexate was significantly lower in patients with good response compared with those with a limited response (Figures 6S,T).

Taken together, in the light of the correlation between ATF3 and DAS-28 score, the relationship between ATF3 expression and methotrexate/tocilizumab response, and the change of ATF3 expression after tocilizumab treatment, we assumed that ATF3 expression in the synovium might promote RA progression.

### Correlation of ATF3 Expression With Gene Set Enrichment Analysis

**FIGURE 7:**
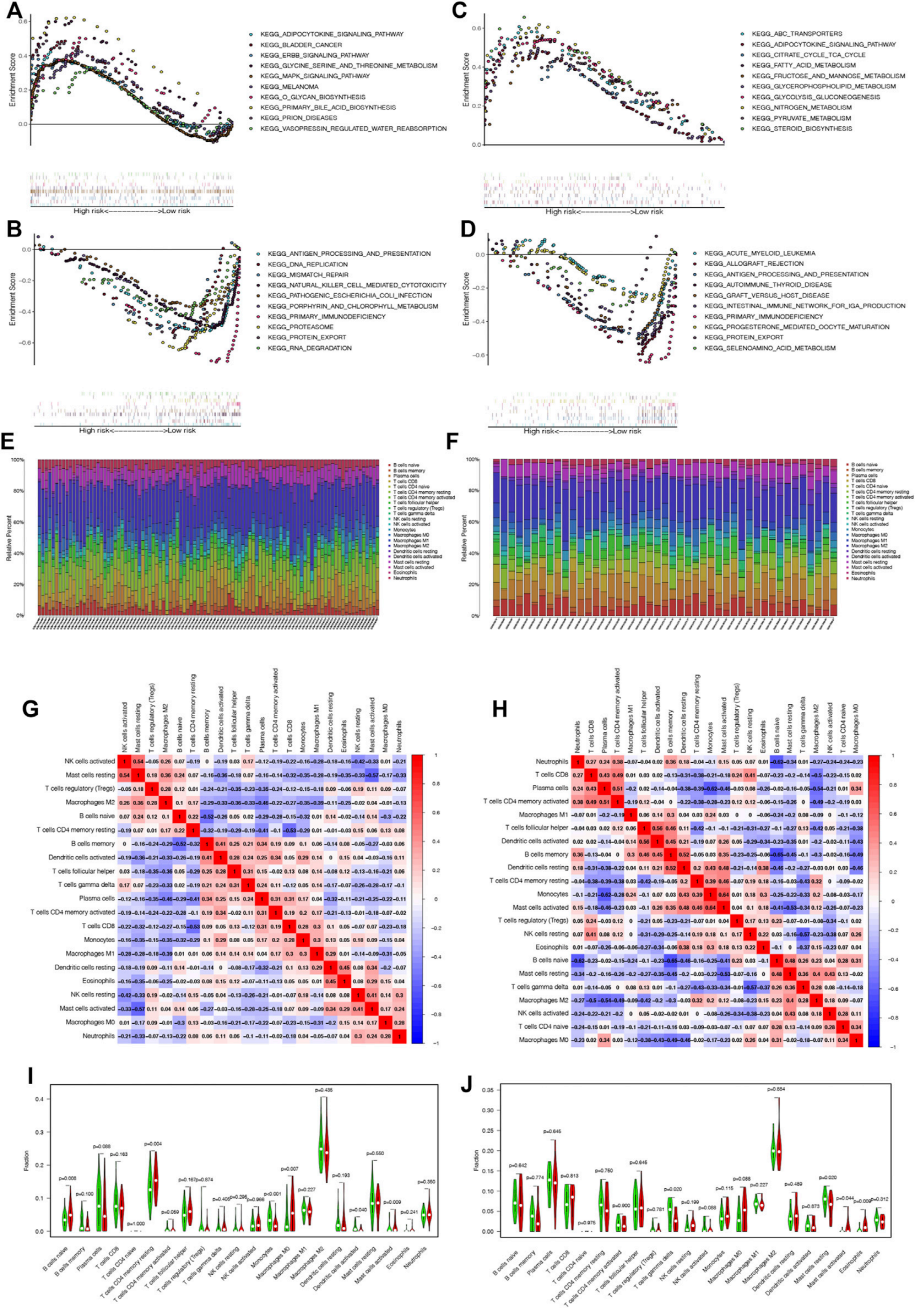
Gene Set Enrichment Analysis shows that pathways enriched in the high ATF3 group were associated with metabolism in the GPL570 and GPL96 databases **(A and C)**, while the inflammation- and immunoregulation-associated pathways were enriched in the low ATF3 group in the GPL570 and GPL96 databases **(B and D)**. **(E and F)** The relative percentage of different infiltrated immune cells in rheumatoid arthritis (RA) samples in the GPL570 and GPL96 databases. **(G and H)** The immune cell correlation matrix was constructed to explore the correlation among all infiltrated immune cells in the GPL570 and GPL96 databases. **(I and J)** The infiltrated immune cells between the high and low ATF3 groups were compared in the GPL570 and GPL96 databases, and the contents of activated mast cells in the high ATF3 group were widely and significantly higher than those in the low ATF3 group in both databases. * *p* < 0.05.

According to the median expression of ATF3, patients in the GPL570 and GPL96 databases can be divided into the high and low ATF3 groups. GSEA showed that these pathways enriched in the high ATF3 group were associated with metabolisms, such as glycine serine and threonine metabolism, O-glycan biosynthesis, primary bile acid biosynthesis in the GPL570 database, and fatty acid metabolism, fructose and mannose metabolism, glycerophospholipid metabolism, and glycolysis gluconeogenesis in the GPL96 database (Figures 7A,C). Similarly, GSEA indicated that KEGG pathways enriched in the low ATF3 group were related to inflammation and immunoregulation, such as antigen processing and presentation, natural killer cell-mediated cytotoxicity, primary immunodeficiency in the GPL570 database, allograft rejection, antigen processing and presentation, and graft versus host disease in the GPL96 database (Figures 7B,D).

### Correlation of ATF3 Expression With Immune Infiltration Characteristics

We applied the CIBERSORT tool to estimate the contents of 22 human immune cells in each RA patient. Figures 7E,F show the relative percentage of different infiltrated immune cells in RA samples in the GPL570 and GPL96 databases. Moreover, the immune cell correlation matrix was constructed, and we noticed that activated NK cells were the most positively correlated with resting mast cells, while resting mast cells were the most negatively correlated with activated mast cells in the GPL570 database. Similarly, monocytes were the most positively correlated with activated mast cells, and memory B cells were the most negatively correlated with naive B cells in the GPL96 database (Figures 7G,H).

Finally, we analyzed and compared the contents of infiltrated immune cells between the high and low ATF3 groups in the GPL570 and GPL96 databases (Figures 7I,J). Interestingly, we discovered that the contents of activated mast cells in the high ATF3 group were widely and significantly higher than those in the low ATF3 group in both databases.

### Validation of ATF3 Promoting Rheumatoid Arthritis Synovial Fibroblast Progression *In Vitro*

#### Infection of Cells With Sh-ATF3 and ATF3-OE

**FIGURE 8:**
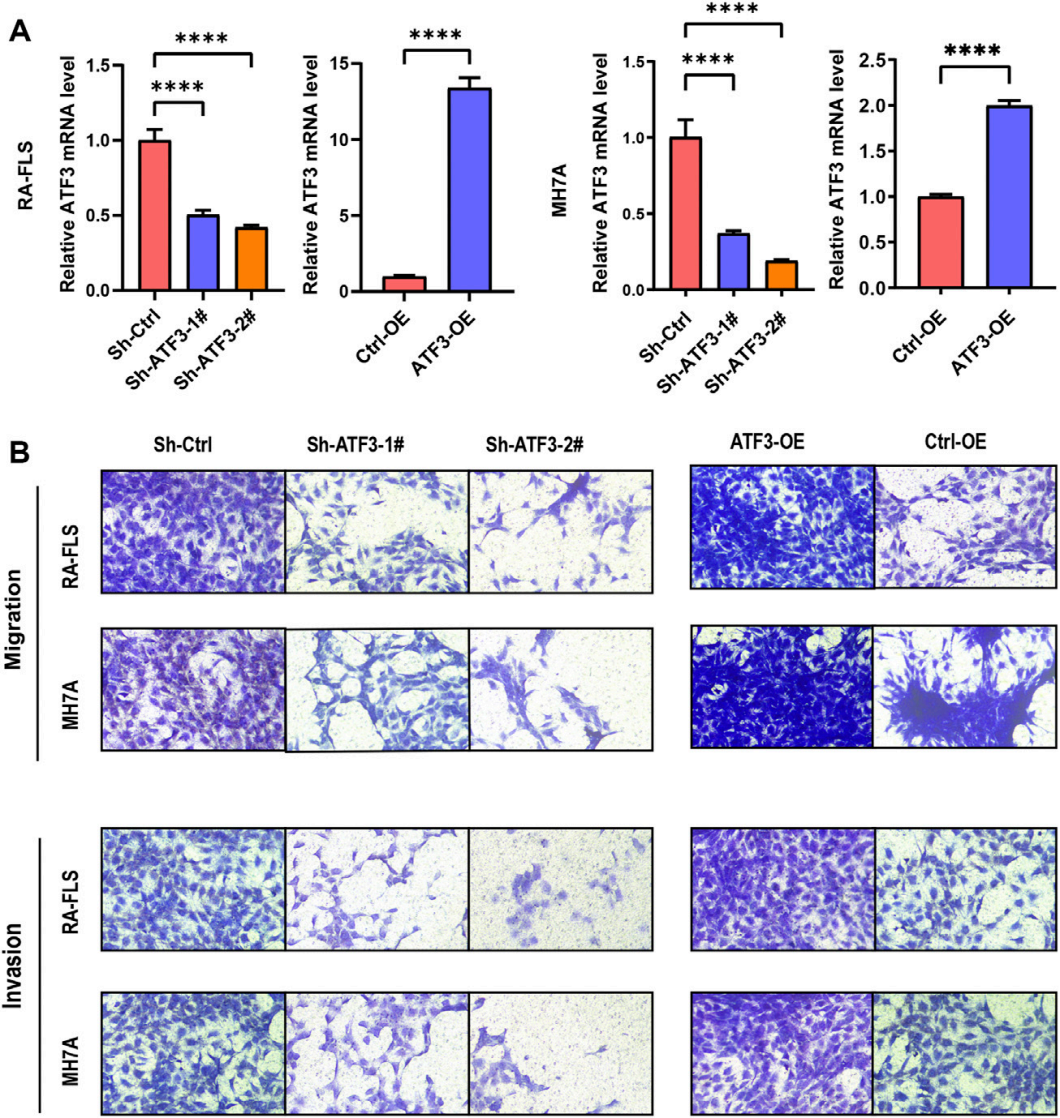
ATF3 could promote cell migration and invasion in rheumatoid arthritis fibroblast-like synoviocyte (RA-FLS) and MH7A. **(A)** The mRNA of knockdown and overexpressed ATF3 was verified by qRT-PCR (mean ± SD, n = 3). **(B)** Transwell assay indicated that ATF3 silence decreased the ability of cell migration and invasion, while ATF3 overexpression significantly promoted cell migration and invasion in RA-FLS and MH7A. *t*-Test was used to analyze the statistical difference. **** *p* < 0.0001.

To further investigate and validate the biological function of ATF3, ATF3 was stably silenced in the Sh-ATF3 groups and overexpressed in the ATF3-OE group. The infection rate was examined by qRT-PCR. Figure 8A shows that ATF3 expression in the Sh-ATF3 groups was dramatically decreased compared with that in the Sh-Ctrl group in RA-FLS and MH7A. Similarly, ATF3 expression in the ATF3-OE group was significantly higher than that in the Ctrl-OE group in RA-FLS and MH7A.

#### ATF3 Promoting Cell Migration and Invasion of Rheumatoid Arthritis Fibroblast-Like Synoviocyte and MH7A

The transwell migration assay showed that knockdown of ATF3 could dramatically decrease cell migration, and overexpression of ATF3 significantly promoted cell migration in RA-FLS and MH7A (Figure 8B). Moreover, the transwell invasion assay also indicated that the invasion ability decreased significantly in ATF3-silenced cells, while the invasion ability was promoted dramatically in ATF3-overexpressed cells (Figure 8B).

#### ATF3 Promoting Proliferation and Inhibiting Apoptosis in Rheumatoid Arthritis Fibroblast-Like Synoviocyte and MH7A

**FIGURE 9:**
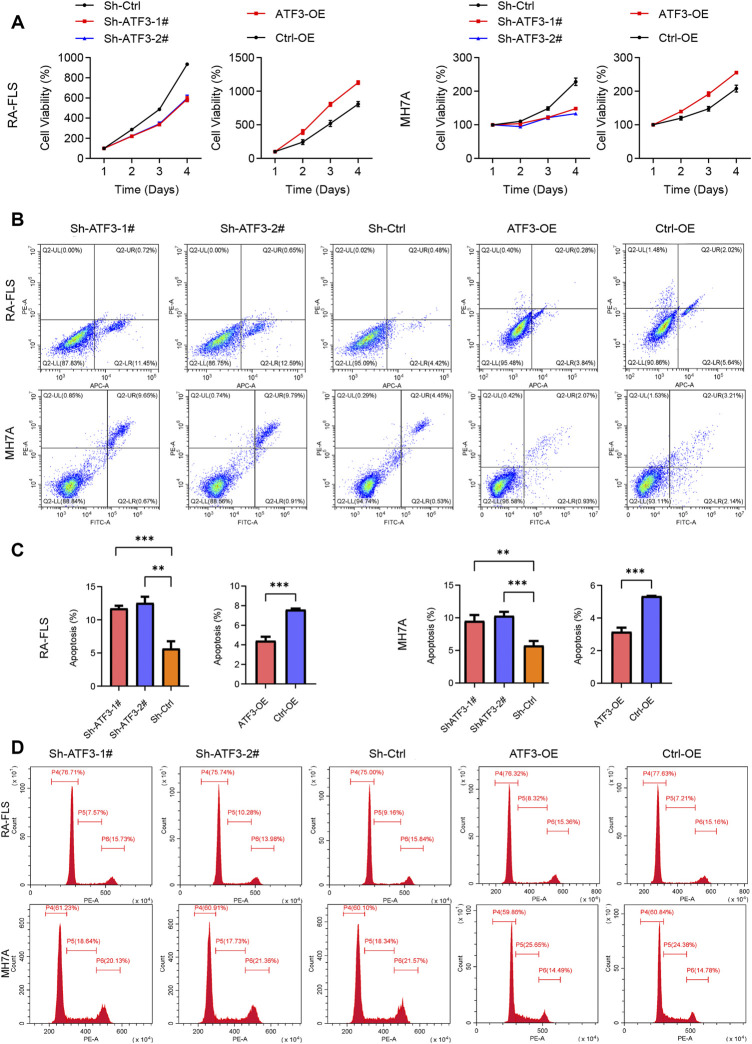
Impact of knockdown and overexpressed ATF3 on cell viability, apoptosis, and cell cycle in rheumatoid arthritis fibroblast-like synoviocyte (RA-FLS) and MH7A. **(A)** The results of Cell Counting Kit-8 (CCK-8) assay revealed that the cell viability in the Sh-ATF3 group was lower than in Sh-Ctrl group, and those in the ATF-OE group were higher than in the Ctrl-OE group (mean ± SD, n = 3). **(B)** Flow cytometry was performed to analyze cell apoptosis. The typical image of apoptosis in RA-FLS and MH7A. **(C)** The results of apoptosis rate showed that Sh-ATF3 induced apoptosis in RA-FLS and MH7A, while ATF3-OE inhibited apoptosis in them (mean ± SD, n = 3). **(D)** Flow cytometry was performed to analyze cell cycle. The typical image of cell cycle in RA-FLS and MH7A. *t*-Test was performed to analyze the statistical difference. ** *p* < 0.01, *** *p* < 0.001.

The effect of ATF3 alteration on the cell viability was detected by CCK-8 assay. The cell viability of the Sh-ATF3 groups was significantly lower than that of the Sh-Ctrl group. Similarly, compared with that of the Ctrl-OE group, the cell viability of the ATF3-OE group was significantly higher in RA-FLS and MH7A (Figure 9A).

Furthermore, we also detected the apoptosis of Sh-ATF3, Sh-Ctrl, ATF3-OE, and Ctrl-OE groups in RA-FLS and MH7A by flow cytometry and found that the proportion of apoptotic cells in the Sh-ATF3 groups was higher than that in the Sh-Ctrl group, while compared with that in the Ctrl-OE group, the proportion of apoptotic cells in the ATF3-OE group was slightly decreased (Figures 9B,C).

#### Silencing ATF3 Inducing G0/G1 Phase Arrest and ATF3 Overexpression Inducing More Cells in S Phase

The effect of ATF3 alteration on the cell cycle of RA-FLS and MH7A was evaluated by flow cytometry. There was a slightly higher proportion of cells in the Sh-ATF3 groups in the G0/G1 phase compared with that in the Sh-Ctrl group (Figure 9D). On the other hand, there was more cell population in the ATF3-OE group enriched in the S phase than that in the Ctrl-OE group (Figure 9D). This indicated that ATF3, as a transcription factor, could promote DNA synthesis to a certain extent.

#### ATF3 Facilitating Pro-Inflammatory Cytokine Secretion in Rheumatoid Arthritis Fibroblast-Like Synoviocyte and MH7A

**FIGURE 10:**
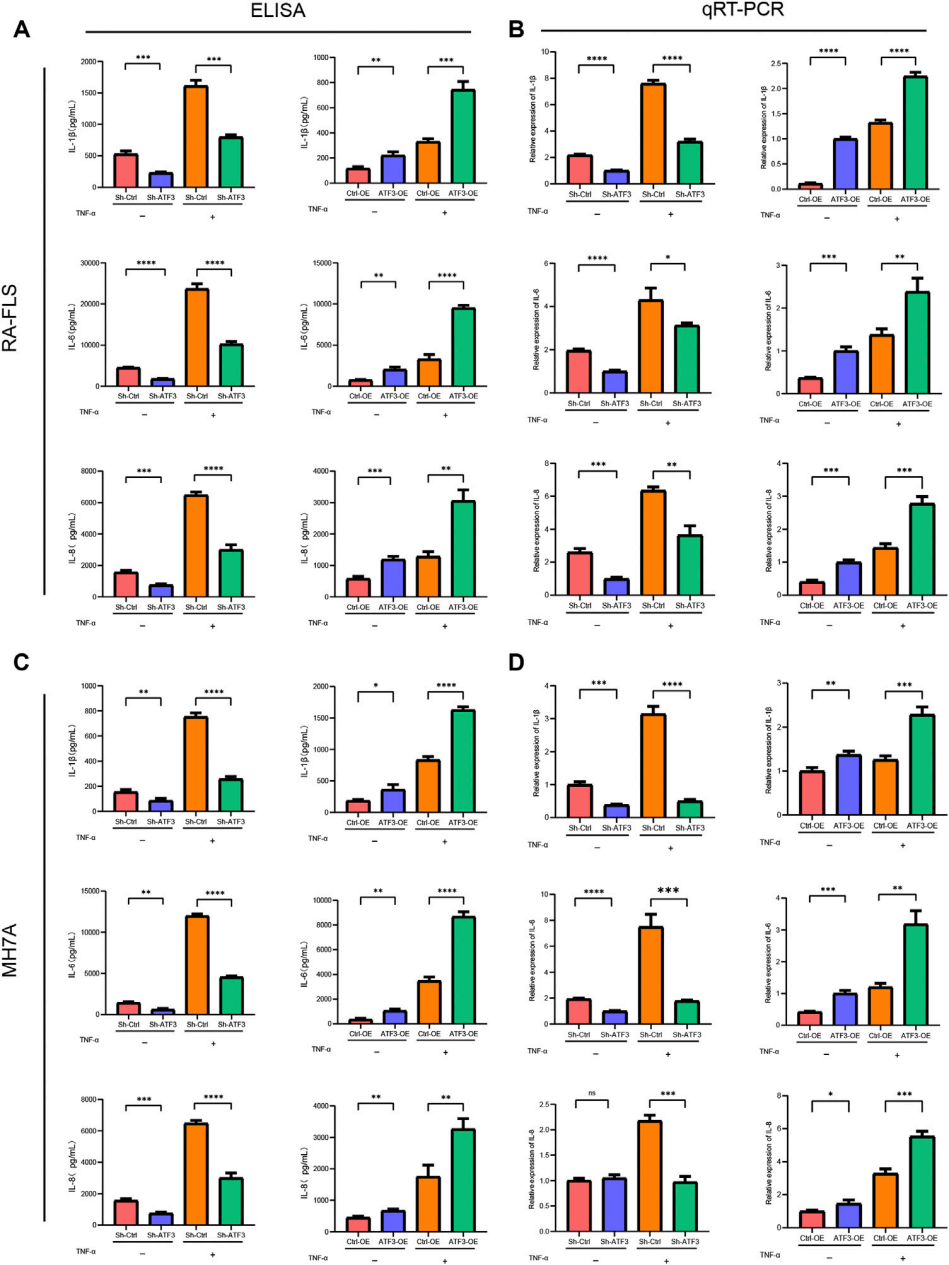
ATF3 facilitating pro-inflammatory cytokine secretion in rheumatoid arthritis fibroblast-like synoviocyte (RA-FLS) and MH7A. **(A and C)** ELISA assays showed that IL-1β, IL-6, and IL-8 in the Sh-ATF3 group were lower than those in the Sh-Ctrl group with or without TNF-α stimulation, which in the ATF3-OE group were higher than those in the Ctrl-OE group (mean ± SD, n = 3). **(B and D)** Similarly, qRT-PCR assays revealed that IL-1β, IL-6, and IL-8 in the Sh-ATF3 group were lower than those in the Sh-Ctrl group with or without TNF-α stimulation, which in the ATF3-OE group were higher than those in the Ctrl-OE group (mean ± SD, n = 3). * *p* < 0.05, ** *p* < 0.01, *** *p* < 0.001, **** *p* < 0.0001.

ELISA and qRT-PCR assay were performed to assess the impact of ATF3 silence and ATF3 overexpression on inflammatory cytokines in the level of protein and mRNA, respectively. Figure 10 shows that IL-1β, IL-6, and IL-8 in the Sh-ATF3 group were lower than those in the Sh-Ctrl group with or without TNF-α stimulation in the level of protein and mRNA. Meanwhile, IL-1β, IL-6, and IL-8 in the ATF3-OE group were significantly higher than those in the Ctrl-OE group, which indicated that ATF3 expression could promote the secretion of inflammatory factors.

## Discussion

RA is a systematically inflammatory and autoimmune disease characterized by pain, swelling, and destruction in local joints, which can limit activities of daily living and lead to physical disability (Smolen et al., 2016; Burmester and Pope, 2017). The FLS, a major cell component maintaining synovial homeostasis, plays a significant role in synovial hyperplasia and inflammation in RA (Nygaard and Firestein, 2020). Inflammation and immune response promote excessive secretion of inflammatory cytokines, growth factors, and MMPs, leading to synovitis and joint degeneration (Wang et al., 2015; Nygaard and Firestein, 2020). Nowadays, a majority of non-tumor necrosis factor inhibitors, including rituximab, infliximab, adalimumab, abatacept, and tocilizumab, were introduced into clinical practice and improved RA patients’ responses and prognoses (Downey, 2016; Moots et al., 2017; Scott, 2017; Gottenberg et al., 2019; Rubbert-Roth et al., 2020; Humby et al., 2021). A prospective cohort study in *BMJ* explored the effectiveness of rituximab, abatacept, and tocilizumab in adult RA patients with poor response to TNF inhibitors, and it was found that compared with abatacept, rituximab and tocilizumab could significantly improve average durations of survival in those refractory RA patients (Gottenberg et al., 2019). Moreover, Humby stratified those refractory RA patients in the light of infiltrating B cells in the synovium and compared the effect of tocilizumab with rituximab in refractory RA patients. Interestingly, he found that tocilizumab is more effective than rituximab for those patients with low B cells in synovial tissue evaluated by RNA-seq, not histopathology (Humby et al., 2021).

Although those DMARDs mentioned above have significantly improved the prognosis of RA patients, approximately 40% of RA patients had a poor response with uncertain mechanisms (Scott, 2017; Gottenberg et al., 2019; Humby et al., 2021). Therefore, it is essential to explore new biomarkers to provide a theoretical possibility for the development of new targeted drugs. It is due to the significant role of FLSs in RA synovium that we downloaded the transcriptome data of RA synovium in the GEO database to explore new biomarkers in our study. WGCNA, a scale-free network algorithm based on gene expression microarray data, is widely used for finding clusters of highly correlated genes in bioinformatics applications (Langfelder and Horvath, 2008; Wan et al., 2018; Tian et al., 2020; Yang et al., 2021), and several studies used this algorithm to explore new biomarkers in RA (Ma et al., 2017; He et al., 2019; Ren et al., 2021). In our study, we applied WGCNA to find the most correlated gene module to RA samples in two databases and intersected the selected gene module with differentially expressed genes to explore potential biomarkers. Finally, ATF3 was screened out and was evaluated via further cell function experiments by *in vitro* and bioinformatics methods.

The PPI plots showed that there was a close relationship among ATF3, ATF4, and JUN. Previous studies once reported that induction of ATF3 transcription by unfolded protein response or amino acid response was mediated by ATF4 and c-Jun recruitment to enhancer elements within ATF3 gene (Hayner et al., 2018). ATF4 induced ATF3 transcription through a promoter-localized C/EBP-ATF response element (CARE) (Fu and Kilberg, 2013). The complex composed of homologous or heterodimers of JUN, FOS, and ATF families was the activator protein-1 (AP-1) molecules of transcription regulator, which were involved in most aspects of cell proliferation, transformation, death, or survival (Shaulian, 2010). Furthermore, Fu (Fu and Kilberg, 2013) found that knockdown of AAR-enhanced c-Jun expression blocked the induction of ATF3 gene, and mutation of either the ATF/CRE or CARE site prevented the c-Jun-dependent increase in ATF3-driven luciferase activity.

ATF3 was activated by various environmental stress signals and was associated with the pathogenesis of various diseases, including cancer, cardiac hypertrophy, infection, and inflammation (Zhao et al., 2016). ATF3 could serve as an oncogene or tumor suppressor gene, which depended on the tumor microenvironment. ATF3 could promote the apoptosis of T-cell lymphoma, multiple myeloma, colon cancer cells, and endometrial cancer cells (Jan et al., 2012; Joo et al., 2015; Wang et al., 2015; Chueh et al., 2017; Wang et al., 2020), while the overexpression of ATF3 promoted the metastasis of breast cancer and prostate cancer (Bandyopadhyay et al., 2006; Wolford et al., 2013). In non-oncological diseases, Wu once reported that pirfenidone could inhibit fibroblast-to-myofibroblast transition via downregulation of ATF3 in RA-associated interstitial lung disease (Wu et al., 2019). Li believed that ATF3 expression in cardiac fibroblast could protect against heart failure, while ATF3 knockout markedly exaggerated hypertensive ventricular remodeling (Li et al., 2017). Moreover, it is reported that ATF3 could also regulate cell growth, apoptosis, invasion, and collagen synthesis in keloid fibroblast through TGF-beta/SMAD signaling pathway (Wang et al., 2021). In our study, we first verified that ATF3 overexpression could promote the proliferation, migration, and invasion of RA-FLS and MH7A (Figures 8, 9) and could stimulate those cells to secrete more pro-inflammation cytokines, like IL-1β, IL-6, and IL-8 (Figure 10). On the other hand, ATF3 silence reduced cell proliferation, migration, and invasion *in vitro* and decreased the secretion of pro-inflammation cytokines. Furthermore, the flow cytometer also indicated that ATF3 overexpression increased the cell proportion in the S phase and decreased the apoptotic cell proportion in RA-FLS and MH7A (Figure 9).

Previous studies have shown that increased proliferation, migration, and invasion of FLSs greatly contribute to RA initiation and progression (Guo et al., 2018). Liu once reported that Sonic Hedgehog (SHH) signaling pathway mediates migration of RA-FLSs via MAPK/ERK pathway and may contribute to the progression of RA (Liu et al., 2018). Choi also revealed that IL-6-regulated Cyr61 is a key player in FLS migration and invasion and eventually contributes to joint destruction in RA (Choi et al., 2020). Similarly, our results also validated that ATF3 overexpression could promote the migration of RA-FLSs, while knockdown of ATF3 reduced the migration ability of FLSs. Moreover, there was also a close relationship between inflammatory cytokines and RA progression. Stromal cells (such as FLSs), antigen-presenting cells (APCs), and macrophages could be locally activated and produce a series of pro-inflammatory factors, which could increase the production of cytokines and synovial vascular leakage and promote the progress of RA under the formation of immune complexes and the activation of complements (Smolen et al., 2018). TNFα is one of the important pro-inflammatory factors that could mediate systemic inflammatory response (Popa et al., 2007). Rossol once illustrated that TNF-α is necessary to trigger the inflammatory response of RA (Rossol et al., 2013). Furthermore, IL-6 is a pro-inflammatory cytokine that triggers host defense by sending out inflammatory signals when microbial infections or tissue damage occur, while the persistence of IL-6 could stimulate the onset of inflammatory and autoimmune diseases such as diabetes and RA (Narazaki et al., 2017). Moreover, the maturation and activation of osteoclasts cause bone erosion via receptor activators of nuclear factor-κB ligand (RANKL) produced by T cells, together with TNF, IL-6, and IL-1 produced by macrophages and FLSs in the synovial lining (Smolen et al., 2018). In our study, ATF3 overexpression could stimulate FLSs to secrete more pro-inflammation cytokines, and ATF3 silence decreases the secretion of pro-inflammation cytokines, which also demonstrated the significant roles of ATF3 in RA progression.

After that, we also investigated the relationships between ATF3 and some pharmacotherapy response parameters, like infliximab response, tocilizumab response, and methotrexate response. First, we found that ATF3 expression in RA synovium before treatment with infliximab seemed to have no significant relationship with infliximab response, but patients with good response accounted for a larger proportion in the high ATF3 group (Figure 6H). Similarly, we analyzed the constitution of patients with different responses in the high and low ATF3 groups and still found that patients with good responses accounted for a larger proportion in the high ATF3 group (Figure 6Q). It is known that infliximab is a monoclonal antibody that specifically blocks TNF-α and improves RA activity. Hu et al. (2017) found that there was an inverse correlation between ATF3 and TNF-α production induced by lipopolysaccharide in mouse monocytes and macrophages pretreated with acute ethanol, and ATF3 may have a novel effect on inhibiting TNF-α induced by lipopolysaccharide. Jeong’s study showed that osteoblasts treated with TNF-α downregulated the osteogenic markers but significantly upregulated the expression of ATF3 (Jeong, 2018). Therefore, there was indeed a negative correlation between ATF3 and TNF-α, which may explain that patients with good responses accounted for a larger proportion in the high ATF3 group. More interestingly, we discovered that ATF3 expression in RA synovium before treatment with tocilizumab or methotrexate could effectively predict pharmacotherapy response, for ATF3 expression in RA patients with a good response was significantly higher than that in patients with a limited response (Figure 6R). Meanwhile, ATF3 expression in RA synovium after treatment with tocilizumab or methotrexate was significantly lower in patients with good response than in those with a limited response (Figures 6S,T). As we have known, tocilizumab is a recombinant humanized monoclonal antibody against human interleukin 6 (IL-6) receptor, and previous studies showed that ATF3 could directly interfere with NF-κB-driven promoters, resulting in decreased expression of inflammatory cytokines, such as IL-6 (Park et al., 2014), which might interpret the ATF3 alteration in RA synovium after treatment with tocilizumab to a certain extent. Although some differences or tendencies mentioned above seemed not statistically significant, these correlations between ATF3 and pharmacotherapy response were instructive and might be verified with a larger sample size and future basic research.

Finally, we investigated the relationships between ATF3 expression and infiltrated immune cells. We discovered that compared with the significantly differentially infiltrated immune cells in the two databases, the contents of activated mast cells were widely and significantly higher in the high ATF3 group than that in the low ATF3 group. Mast cells, a type of tissue-resident innate immune cells, interacted with various immune cells and played a potential pathologic role in various autoimmune disorders (Xu and Chen, 2015; Min et al., 2020). The activated mast cells could secrete cytokines, stimulate other immune cells and local synoviocytes, and recruit circulating inflammatory cells into the RA synovium (Woolley and Tetlow, 2000). Rivellese once reported that high mast cell count is associated with local and systemic inflammation, autoantibody positivity, and high disease activity, and mast cells in early RA positively correlated with disease severity and support B-cell autoantibody production, which were in accordance with the role of ATF3 verified by cell function experiments in our study (Rivellese et al., 2018). Therefore, it is indicated that RA patients with higher ATF3 expression in the synovium seemed to have higher contents of activated mast cells and more serious disease conditions, but they might respond better when receiving the treatment of tocilizumab or methotrexate.

## Conclusion

We successfully identified and validated that ATF3 could serve as a novel biomarker in RA, which correlated with pharmacotherapy response and immune cell infiltration. The high expression of ATF3 had a positive correlation with systemic inflammation and high disease activity.

## Data Availability

Publicly available datasets were analyzed in this study. These data can be found here: https://www.ncbi.nlm.nih.gov/geo/.
